# A Hybrid Nanomaterial Based on Single Walled Carbon Nanotubes Cross-Linked via Axially Substituted Silicon (IV) Phthalocyanine for Chemiresistive Sensors

**DOI:** 10.3390/molecules25092073

**Published:** 2020-04-29

**Authors:** Maxim Polyakov, Victoria Ivanova, Darya Klyamer, Baybars Köksoy, Ahmet Şenocak, Erhan Demirbaş, Mahmut Durmuş, Tamara Basova

**Affiliations:** 1 Nikolaev Institute of Inorganic Chemistry SB RAS, Ak. Lavrentiev Avenue, 3, 630090 Novosibirsk, Russia; polyakov_m.s@mail.ru (M.P.); vikiivanova2660@gmail.com (V.I.); klyamer@niic.nsc.ru (D.K.); 2 Saint Petersburg State University of Architecture and Civil Engineering, Vtoraya Krasnoarmeiskaya, 4, 190005 Saint Petersburg, Russia; 3 Department of Chemistry, Gebze Technical University, Gebze, 41400 Kocaeli, Turkey; baybarsky@gmail.com (B.K.); asenocak@gtu.edu.tr (A.Ş.); erhan@gtu.edu.tr (E.D.); durmus@gtu.edu.tr (M.D.)

**Keywords:** carbon nanotubes, hybrid materials, covalent functionalization, phthalocyanine, gas sensor, chemiresistive sensor

## Abstract

In this work, the novel hybrid nanomaterial SWCNT/SiPc made of single walled carbon nanotubes (SWCNT) cross-linked via axially substituted silicon (IV) phthalocyanine (SiPc) was studied as the active layer of chemiresistive layers for the detection of ammonia and hydrogen. SWCNT/SiPc is the first example of a carbon-based nanomaterial in which an axially substituted phthalocyanine derivative is used as a linker. The prepared hybrid material was characterized by spectroscopic methods, thermogravimetry, scanning and transmission electron microscopies. The layers of the prepared hybrid were tested as sensors toward ammonia and hydrogen by a chemiresistive method at different temperatures and relative humidity as well as in the presence of interfering gases like carbon dioxide, hydrogen sulfide and volatile organic vapors. The hybrid layers exhibited the completely reversible sensor response to both gases at room temperature; the recovery time was 100–200 s for NH_3_ and 50–120 s in the case of H_2_ depending on the gas concentrations. At the relative humidity (RH) of 20%, the sensor response was almost the same as that measured at RH 5%, whereas the further increase of RH led to its 2–3 fold decrease. It was demonstrated that the SWCNT/SiPc layers can be successfully used for the detection of both NH_3_ and H_2_ in the presence of CO_2_. On the contrary, H_2_S was found to be an interfering gas for the NH_3_ detection.

## 1. Introduction

To date, a large variety of materials on the basis carbon nanotubes (CNT) has been obtained, among them ordered layers [1], nanocarbon fibers [2,3], bucky paper [4,5], modified electrodes [6,7,8,9], gas or cation absorbers [10,11] and some others [12]. Pristine carbon materials can be modified by various ways, e.g., through heteroatom doping [13], covalent attachment of different atoms [14,15,16], functional groups [17,18,19,20] or molecules [21,22] as well as by non-covalent adsorption of various polyaromatic molecules [23,24,25,26], nanoparticles [4,27] or polymers [28].

One of the practical aspects of such hybrid materials is their use as the active layers of chemiresistive gas sensors. It is well known that the conductivity of different types of pristine CNTs is determined by their symmetry and structure [29]. In 2000 Kong et al. [30] firstly discovered that the conductivity of semiconductor carbon nanotubes changed when different molecules were adsorbed on their surface and that property could be used in gas sensor devices. Thus, strong oxidants—electron acceptors (e.g., NO_2_)—significantly increase conductivity of p-type semiconductor CNTs due to the enhancement of the hole concentration. On the contrary, reducing gases, which are electron donors, lead to a decrease in conductivity due to the recombination of electrons and holes. Nowadays, the sensor characteristics of CNT-based hybrid materials toward a variety of gases, such as NH_3_, NO_2_, CH_4_, SO_2_, H_2_S, etc., and volatile organic compounds (ethanol, acetone, and chloroform) have been studied [17,23,31,32,33]. It has also been shown that CNT-based hybrid materials show noticeably higher sensor response and better sensing performance in comparison with pristine carbon nanotubes.

Metallophthalocyanines are also widely used as active layers of gas sensors due to their ability to change their conductivity in the presence of different gases by the force of charge transfer processes [34,35]. There are several examples in the literature on the applications of hybrid materials created on the basis of CNTs and MPcs as active layers of chemical sensors [23,32,36]. In our previous studies, hybrid materials were obtained using both covalent and non-covalent functionalization of CNT surface by phthalocyanine molecules [37]. It has been shown that the hybrid materials exhibited better sensor characteristics than pristine nanotubes owing to the combination of CNT properties such as conductivity and developed surface area with the addition of metal phthalocyanines, viz. their high sensor response to the gaseous analytes and ability to form ordered thin films. 

Creation of new porous cross-linked or three-dimensional (3D) carbon nanostructures is another important fundamental task of nanotechnology. Some structures of this type of materials are of practical interest for the development of a new generation of functional carbon materials. 3D structures formed via covalent bonding between CNT building blocks are described in the literature [38,39,40,41]. One of the important approaches to obtain 3D structures of nanotubes is their functionalization by boronic acid derivatives according to the Pd-catalyzed Suzuki coupling reaction [42]. Another approach to covalent bonding of CNTs is the Pd-catalyzed Sonogashira coupling reaction, viz. a cross-coupling reaction between vinyl and aryl halides with terminal alkynes, catalyzed by palladium and copper [43]. Apart from this, covalent CNT cross-linking by different organic molecules (linkers) are also described for the creation of 3D-carbon materials. For instance, the covalent binding of CNT via 1,4-diethylbenzene and 4,4′-diethynyldiphenyl linkers was carried out by the Sonogashira reaction [44]. It was also shown [45,46,47] that 3D hybrid materials of single walled carbon nanotubes (SWCNT) with organic molecules could be obtained by the azide-alkyne cycloaddition reaction. In practical terms 3D-materials are usually considered as gas absorbers with high surface area [41,44,48]. Cai et al. [49] synthesized 3D porous carbon material and researched its electrocatalytic properties. The obtained material exhibited an excellent catalytic activity in oxygen reduction reactions. A rechargeable Zn-air battery based on the obtained 3D porous catalyst demonstrated a high peak power density of 119 mW·cm^−2^ at the cell voltage of 0.578 V while retaining excellent stability over 250 charge-discharge cycles. At the same time, porous CNT-based 3D hybrid structures where phthalocyanines are used as linking molecules have not yet been described in the literature. 

**Figure 1:**
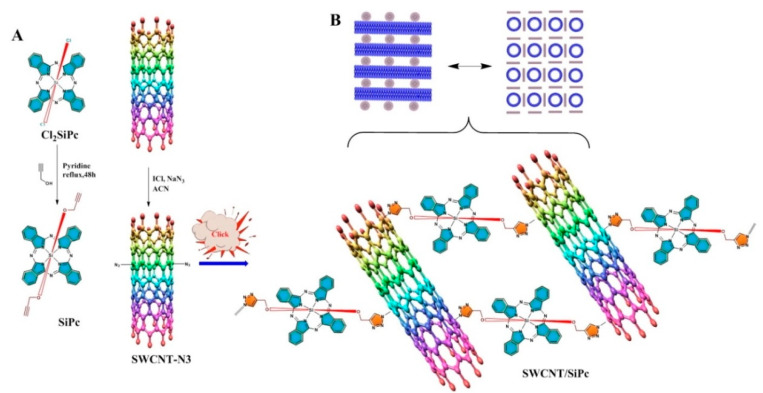
(**A**) Synthetic pathway of axially terminal propynoxy substituted silicon (IV) phthalocyanine and its hybrid nanomaterial with SWCNT; (**B**) Schematic illustration of the SWCNT/SiPc hybrid material.

In this work, the hybrid material of SWCNTs cross-linked via axially substituted silicon (IV) phthalocyanine (SiPc) (Figure 1) was synthesized and characterized by spectral methods (such as Fourier Transform Infrared (FT-IR), Raman, Ultraviolet-Visible absorption (UV-Vis) spectroscopies) and thermogravimetry. The surface morphology of this hybrid material was monitored by scanning electron microscopy (SEM) and transmission electron microscopy (TEM). The layers of this hybrid material were tested as sensors toward ammonia and hydrogen gases by a chemiresistive method. The novelty of this work is to create a new material for fast, accurate and selective detection of these gases. Detection and reduction of ammonia emissions is one of the most important and difficult tasks in agricultural industry [50,51]. On the other hand, NH_3_ is a gas biomarker of certain kidney diseases, and the accurate quantification of ammonia concentration (less than 5 ppm) in exhaled human breath can provide additional diagnostics liver and kidney disfunction [52]. Hydrogen is a highly flammable gas and will burn at concentrations as low as 4% in air. Rapid and accurate detection of hydrogen is necessary during the production, storage, and use of hydrogen, and is also necessary for controlling its concentration in nuclear reactors, coal mines, and semiconductor manufacturing, etc. [53]. 

## 2. Results and Discussion

### 2.1. Synthesis and Characterization

For the creation of SWCNT/SiPc hybrid, the novel axially bis(propynoxy)substituted silicon (IV) phthalocyanine (SiPc) was synthesized by the reaction of dichlorosilicon (IV) phthalocyanine with propargyl alcohol in pyridine. The SWCNT/SiPc hybrid material is the first example of the three-dimensional carbon nanotubes cross-linked via axially substituted phthalocyanines. After purification, the structure of this novel phthalocyanine was confirmed by spectroscopic methods such as UV-Vis, ^1^H-NMR, FT-IR, as well as by mass spectrometry and elemental analysis. In the FT-IR spectrum of the SiPc, the vibration band belonging to Si–O–C was observed at 1081 cm^−1^. The characteristic vibration bands of C≡C-H and C≡C were observed at 3261 and 2110 cm^−1^, respectively [54]. The other main vibrations of aromatic-CH and aliphatic-CH groups were monitored at 3010 and 2957–2852 cm^−1^, respectively. In the ^1^H-NMR spectrum of SiPc, the aromatic H_α_ and H_β_ protons of phthalocyanine ring were observed between 9.68–9.66 and 8.37–8.36 ppm, respectively [55]. The aliphatic protons in Si–O–CH_2_ and C≡C-H groups were shifted to the negative region due to the magnetic anisotropy behavior of silicon (IV) phthalocyanine ring. These protons were observed at −0.99 ppm for CH_2_ and 0.63 ppm for C≡C-H as singlets. In the MALDI-TOFF mass spectrum of this phthalocyanine, the molecular ion peak was observed at 650.66 *m*/*z* as [M]^+^ and 595.43 *m*/*z* as the [M-OCH_2_CCH]^+^ fragment, and this result was compatible with the theoretical molecular weight of this compound [56].

**Figure 2:**
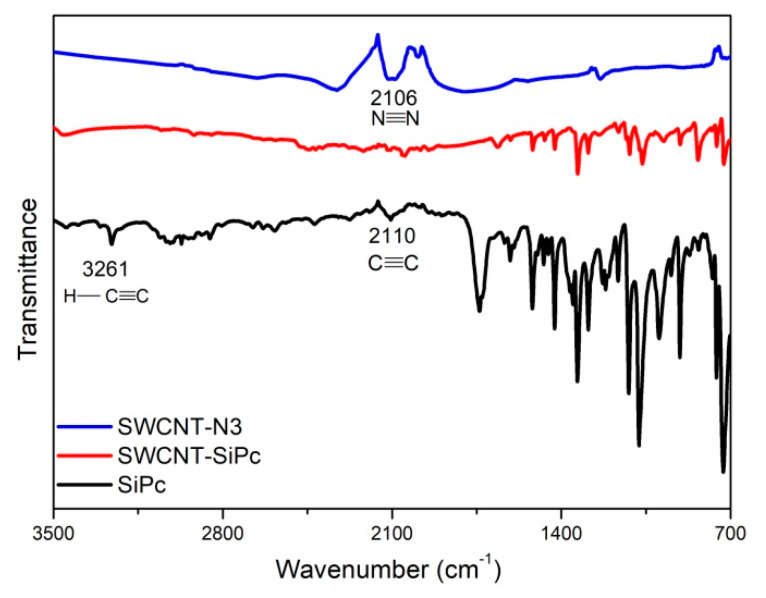
FT-IR spectra of SiPc, SWCNT-N3 and SWCNT/SiPc hybrid.

FT-IR spectra of the silicon (IV) phthalocyanine substituted with terminal propynoxy groups in axial positions (SiPc), SWCNTs bearing azido groups (SWCNT-N3) and their hybrid nanomaterial (SWCNT/SiPc) are shown in Figure 2. The vibrational peaks corresponding to ethynyl groups in the SiPc derivative (observed at 3261 and 2110 cm^−1^) and azide groups in SWCNT-N3 (observed at 2106 cm^−1^) almost disappear in the FT-IR spectrum of SWCNT/SiPc hybrid which indicates that the “Click” reaction was occurred between SiPc and SWCNT-N3 (Figure 2). At the same time, several peaks belonging to phthalocyanine moiety are observed in the FT-IR spectrum of the hybrid, viz. 2990–2835 (Aliphatic CH), 1608, 1518 (C=C), 1135 (Ar C=C) and 1122 cm^−1^ (Si-O). A small peak corresponding to azide vibration was still observed in the FT-IR spectrum of SWCNT/SiPc due to the rest of the azide groups in the hybrid nanomaterial.

**Figure 3:**
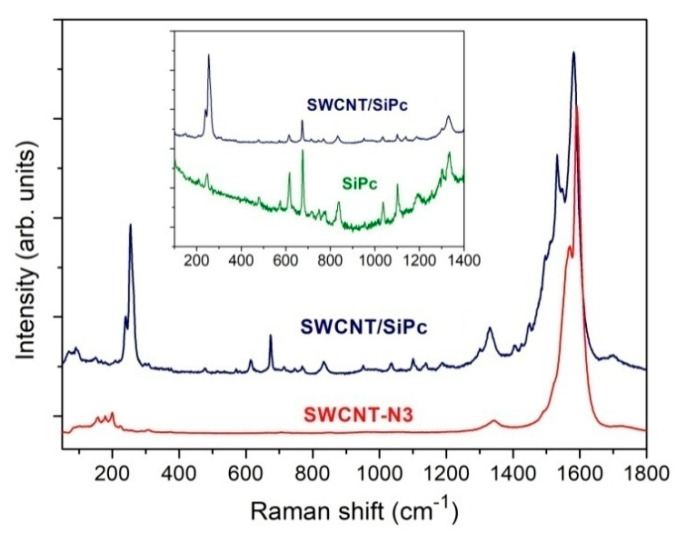
Raman spectrum of SWCNT/SiPc hybrids in comparison with that of SWCNT-N3. The inset shows the enlarged spectrum of SWCNT/SiPc in the range from 150–1400 cm^−1^ in comparison with that of SiPc.

The Raman spectrum of SWCNT/SiPc is compared to those of SiPc and SWCNT-N3 in Figure 3. The D and G bands in the spectrum of SWCNT-N3 are located at 1343 and 1590 cm^−1^, respectively, while their positions shifted to 1332 and 1582 cm^−1^ after the formation of the hybrid. The ratio of intensities of D and G bands (I_D_/I_G_), which is usually used for the characterization of functionalization of carbon nanomaterials [57], increases more than twice, namely, the I_D_/I_G_ ratio in the spectrum of SWCNT-N3 is 0.05, while in the spectrum of SWCNT/SiPc hybrid is 0.12. 

This value is less than that observed in the case of SWCNT hybrids obtained by non-covalent functionalization by phthalocyanine molecules (e.g., the I_D_/I_G_ ratio was 0.052 in the case of SWCNTs non-covalently modified with tetrasubstituted ZnPc [58]), but closer the I_D_/I_G_ values obtained for SWCNTs covalently modified with other polyaromatic molecules (e.g., 0.092 in the case of 3D hybrids of SWCNT cross-linked via phenylcoumarin molecules [57]). The intensity of radial breathing modes (RBM) observing in the spectrum of SWCNT-N3 in the range from 150 to 230 cm^−1^ becomes lower in the SWCNT/SiPc spectrum. RBMs correspond to radial expansion-contraction of the nanotube and, their frequencies depend on the nanotube diameter. A decrease of their intensities in the spectrum of SWCNT/SiPc hybrid may relate to the change of electronic structure of SWCNT after the formation of covalent bonds with SiPc molecules and a cross-linked structure. Apart from this, bands corresponding to the vibration of phthalocyanine moiety are clearly observed in the spectrum of SWCNT/SiPc hybrids (see inset to Figure 3).

**Figure 4:**
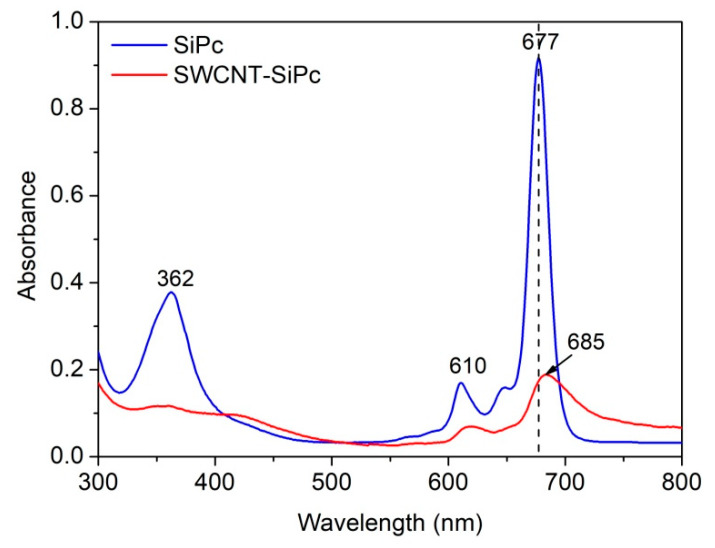
UV-Visible electronic absorption spectra of SiPc and its SWCNT/SiPc hybrid nanomaterial in DMF.

Figure 4 depicts the UV-Visible absorption spectra of the SiPc solution in DMF and the suspension of its hybrid nanomaterial SWCNT/SiPc obtained by sonication in DMF. SiPc shows characteristic absorption bands at 677 and 362 nm in DMF known as Q and B bands, respectively. The vibronic satellite at 610 nm is also observed. The intensities of these bands are lower in the spectrum of SWCNT/SiPc hybrid because of the lower amount of SiPc molecules attached to the surface of carbon nanotubes compared to the SiPc solution. 

In the electronic absorption spectrum of SWCNT/SiPc hybrid, the Q band was observed as a broad peak with the absorption maximum at 685 nm, bathochromically shifted compared to that in the electronic absorption spectrum of SiPc. The shift to the higher wavelength might be ascribed to the strong electronic interaction between SWCNTs and SiPc derivative in the SWCNTs/SiPc hybrid (MPc is usually regarded as an electron donor, while SWCNTs as an electron acceptor) [59], which reduces the aggregation of the hybrids and hence changes the absorption. 

### 2.2. Thermogravimetric Analysis

**Figure 5:**
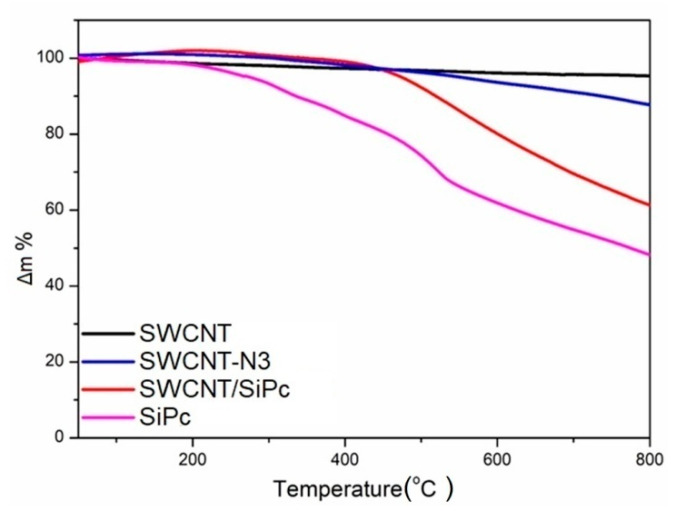
Thermogravimetric analysis of SWCNT/SiPc hybrid nanomaterial in comparison with pristine SWCNTs, SWCNT-N3 and SiPc.

The amount of SiPc molecules covalently bonded to SWCNT was calculated using TGA data (Figure 5). The weight losses of SiPc, pristine SWCNT, SWCNT-N3 and SWCNT/SiPc at 800 °C were found to be 51.70%, 4.60%, 12.17% and 38.66%, respectively. The weight losses due to functional chemical groups in SWCNT were determined as 7.57% for SWCNTs-N3 and 26.49% for SWCNT/SiPc. The number of azide groups on SWCNTs-N3 was calculated according to the literature [47,60,61] to be 1 per 43 carbon atoms. The weight loss due to elimination of the SiPc fragment from SWCNT/SiPc hybrids was 51.23% (26.49%/51.70%). The amount of SiPc covalently bonded to SWCNT was estimated in the same way to be one SiPc molecule per 57 carbon atoms in the hybrid according to the calculations (51.23% × 650.16)/(48.77% × 12). 

The amount of SiPc molecules in this hybrid is about four times larger than that in SWCNT-based hybrid obtained by covalent functionalization with other phthalocyanine molecules [36,37] and comparable with the amount of other linking molecules, viz. pyrene and coumarin, in similar cross-linked hybrid materials [45,61].

### 2.3. Morphological Characterization of the Hybrid Material

**Figure 6:**
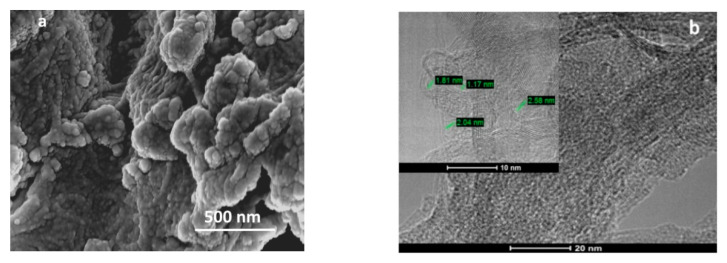
(**a**) SEM and (**b**) TEM images of the SWCNT/SiPc hybrid material.

The morphology of the SWCNT/SiPc hybrid was studied by SEM (Figure 6a) and TEM (Figure 6b). The SWCNT/SiPc hybrid consists of aggregates of tightly packed cross-linked nanotubes. The TEM image of SWCNT/SiPc (Figure 6b) demonstrates a structure of cross-linked carbon nanotubes with pores of diameter 1–2.6 nm. Such a porous structure makes the 3D material a good candidate for gas sensor applications.

### 2.4. Sensor Properties of SWCNT/SiPc Hybrid Material

#### 2.4.1. Sensor Response of SWCNT/SiPc Layers to Ammonia and Hydrogen

**Figure 7:**
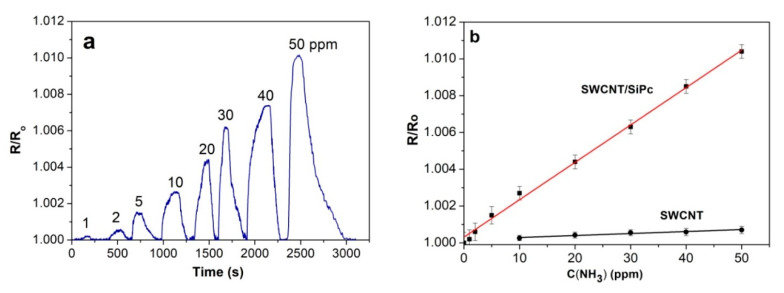
(**a**) Sensor response of SWCNT/SiPc layers toward NH_3_ (1–50 ppm) measured at RH 5% and 25 °C; (**b**) dependence of the sensor response of SWCNT/SiPc and pristine SWCNT on ammonia concentration.

**Figure 8:**
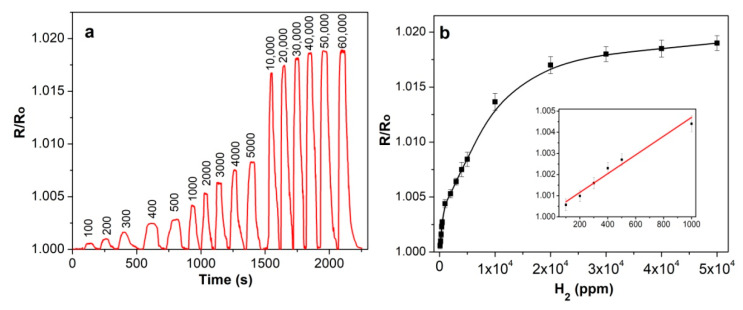
(**a**) Sensor response of SWCNT/SiPc layers toward H_2_ (100–60,000 ppm) measured at RH 5% and 25 °C; (**b**) Dependence of the sensor response on hydrogen concentration.

The chemiresistive sensor response of the hybrid layers was studied toward reducing gases, viz. NH_3_ and H_2_. Figure 7 and Figure 8 show the relative sensor responses of SWCNT/SiPc toward ammonia and hydrogen, respectively. The investigated NH_3_ concentration varied from 1 to 50 ppm because this is within the range of its maximum permissible concentration, which is important for practical applications. 

The concentration of hydrogen was changed from 100 to 60,000 ppm. Sensor response is given as R/R_o_ where R is the resistance at the detected concentration of analyte and R_o_ is the initial layer resistance. SWCNT/SiPc layers exhibit a completely reversible sensor response to ammonia and hydrogen in the investigated concentration range at room temperature. The injection of both ammonia and hydrogen into the flow cell leads to an increase of the resistance of SWCNT/SiPc layer. Similar behavior was also observed for SWCNTs and their hybrids with other molecules [62]. The gas detection mechanism is based on a change in the material conductivity, which is the result of the reaction of the gas molecules with the CNT surface. Direct charge transfer between the analyte and CNTs was identified as the main sensing mechanism for polar analytes. Under ambient conditions, nanotubes are p-type semiconductors as a result of physical adsorption of oxygen molecules on their surfaces [63]. Thus, interaction with electron donor molecules such as ammonia leads to the depletion of holes level in the investigated layer [64]. The interactions that occur between surface functional groups and NH_3_ molecules are weak, such as hydrogen bonds, electrostatic interactions (dipole-dipole interactions), which causes the reversibility of the response [65]. 

It is necessary to mention that the sensor response to hydrogen is much less than that to ammonia at the same concentration. This may be connected with the better electron donating ability of NH_3_ in comparison with H_2_ and/or different nature of interaction of the sensing layer with hydrogen. It is worth mentioning that the sensor response of SWCNT/SiPc to ammonia is 10 times higher than that of pristine SWCNTs studied in our previous works [37,66]. The recovery time was 100–200 s in the case of NH_3_ and 50–120 s in the case of H_2_ depending on the gas concentrations.

The change in the resistance of hybrid films when interacting with H_2_ appears to have different mechanism than in the case of ammonia. It was assumed in the literature [67,68] that atmospheric oxygen absorbs at the air/hybrid interface. The formation of charge-transfer complexes at the interface between air and phthalocyanine attached to the SWCNT walls leads to the generation of oxidized MPc^+^ and O_2_^−^ species and injection of hole charge carriers into the layer’s bulk. Then the sensor layer is exposed to hydrogen atmosphere, and the hydrogen molecules react with the adsorbed oxygen species. The redox reaction is exothermic and leads to the fast desorption of produced H_2_O molecules from the surface. Due to the released electrons, a change of the resistance of the hybrid layer is observed.

The dependence of sensor response of SWCNT/SiPc to ammonia concentration is almost linear in the investigated concentration range (Figure 7b). The sensor response to hydrogen was investigated in the wider concentration range up to 50,000 ppm because hydrogen is a highly flammable gas and it burns at concentrations as low as 4% in air and its detection at comparatively high concentrations in air is also of practical importance. The layer exhibits linear dependence between 100 ppm and 1000 ppm of hydrogen (Figure 8b). For the concentrations above 1000 ppm the response is no more linear and the sensitivity to hydrogen continuously decreases. This means that the sensors reach a saturation state. The limits of detection (LOD) were calculated according to the 3*·s/m* [69], where *m* is the slope of the calibration plots (Figure 7b and Figure 8b) and *s* is the standard deviation of the response value at the NH_3_ concentration of 1 ppm and H_2_ concentration of 100 ppm. The standard deviation of the response value was calculated using the data for five parallel samples. These values were 0.5 ppm and 70 ppm for ammonia and hydrogen, respectively. 

Characteristics of the investigated SWCNT/SiPc-based sensor are compared with those of some sensors described in the literature in Table 1. The sensing layers based on SWCNT/SiPc are quite competitive with the active layers on the basis of such carbon nanomaterials as graphene (G), reduced graphene oxide (rGO), multiwalled carbon nanotubes (MWCNT) functionalized with metal oxides, phthalocyanines, poly(3,4-ethylenedioxythiophene) polystyrene sulfonate (PEDOT-PSS), described in the literature. The SWCNT/SiPc layers exhibit a reversible sensor response at room temperature, a low detection limit, and low values of response and recovery times, compared with the other sensors.

#### 2.4.2. Effect of the Humidity

**Figure 9:**
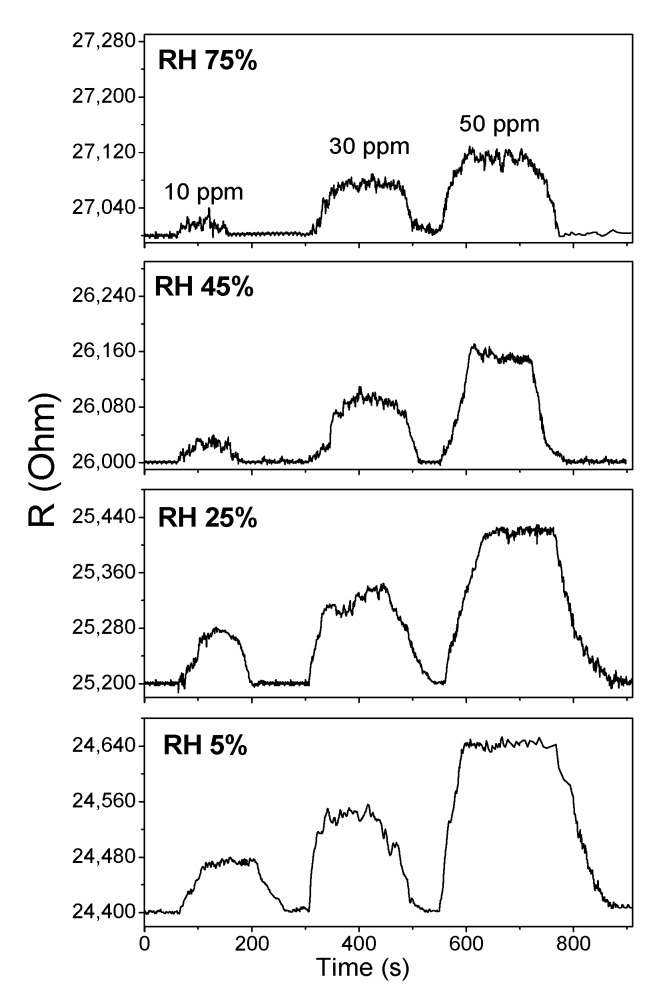
Sensor response of SWCNT/SiPc hybrid layers toward NH_3_ (10, 30 and 50 ppm) measured at RH 5, 25, 45 and 75% and 25 °C.

Water vapor often acts as an interfering gas for the detection of electron donor gases. Therefore, the effect of water on the sensor response is of great interest for this study. The sensor responses of SWCNT/SiPc hybrids toward NH_3_ (1–50 ppm) measured at RH 5, 25, 45 and 75% are shown in Figure 9 as examples. At RH 20% the sensor response was almost the same as that measured at RH 5%, whereas the further increase of RH to 50 and 70% led to its 2–3 fold decrease. At the same time, the increase of RH does not noticeably influence the response and recovery times.

Like the NH_3_ molecule, water is also an effective electron donor agent. The presence of water leads to the increase of the base resistance of hybrid layers from 2.4·10^4^ to 2.7·10^4^ Ohm (Figure 9). Sorption of water molecules on the surface of hybrid material appears to lead to the occupation of active sites and contribute to the saturation of the sensing layer. Consequently, the response to ammonia decreases with the increase of RH [64]. 

#### 2.4.3. Cross-Sensitivity toward Various Gases

**Figure 10:**
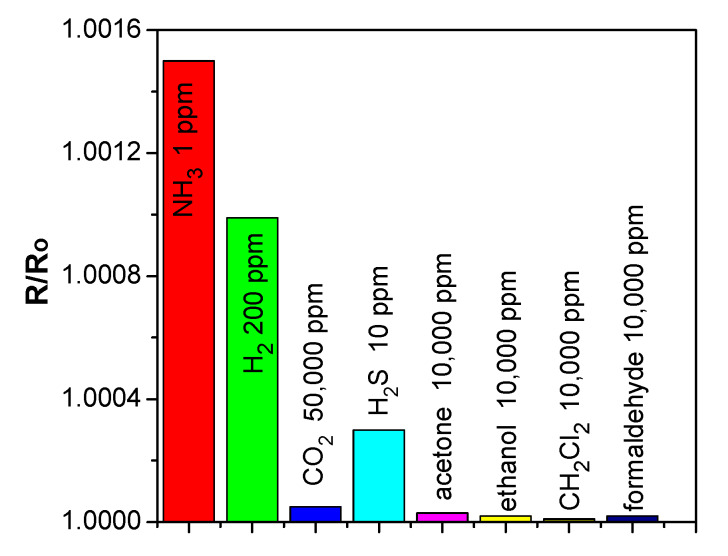
Sensor response of SWCNT/SiPc hybrid layers to different analytes.

The selectivity and cross-sensitivity of the SWCNT/SiPc hybrid material was tested using H_2_S, CO_2_, acetone, dichloromethane, ethanol, and formaldehyde. The diagram of the sensor response of SWCNT/SiPc hybrid layers toward NH_3_ and these interfering analytes is shown in Figure 10. The sensitivity of SWCNT/SiPc layers to ammonia is noticeably higher compared to that towards other investigated gases and volatile organic vapors (VOC). This makes this hybrid material attractive for the detection of ammonia in the presence of VOCs and CO_2_. 

**Figure 11:**
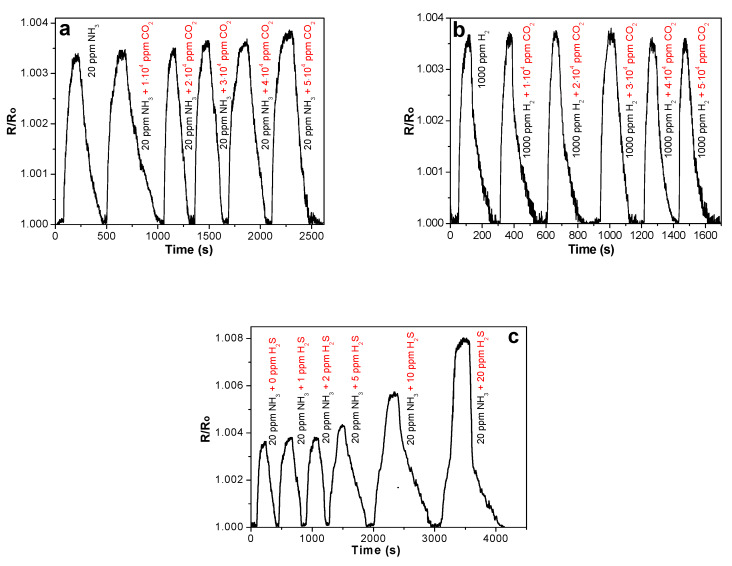
Sensor response of SWCNT/SiPc toward (**a**) ammonia (20 ppm) and (**b**) hydrogen in the presence of CO_2_ (from 1∙10^4^ to 5∙10^4^ ppm) and toward ammonia in the presence of H_2_S (from 1 to 20 ppm) **(c)** measured at RH 5% and 25 °C.

The detection of ammonia and hydrogen was also carried out in the mixture with H_2_S and CO_2_. Figure 11a shows the sensor response of SWCNT/SiPc to NH_3_ (20 ppm) in presence of CO_2_ (1·10^4^–5·10^4^ ppm). The addition of comparatively high concentrations of CO_2_ does not lead to any noticeable changes in the sensor response value of SWCNT/SiPc layers to 20 ppm NH_3_. The value of sensor response to hydrogen is also stable and does not change upon addition of carbon dioxide (Figure 11b). It means that both ammonia and hydrogen can be successfully detected in the presence of CO_2_. 

On the contrary, H_2_S was found to be an interfering gas for the NH_3_ detection. Figure 11c shows that while low concentrations of H_2_S (below 5 ppm) does not affect the sensor response to ammonia (20 ppm), the further growth of H_2_S concentrations in the investigated gas mixture causes a noticeable increase in the sensor response. The electron donating character of H_2_S similarly to NH_3_ appears to cause the additional increase of the layer resistance and, as a consequence, the total sensor response. 

#### 2.4.4. Effect of Operating Temperature

**Figure 12:**
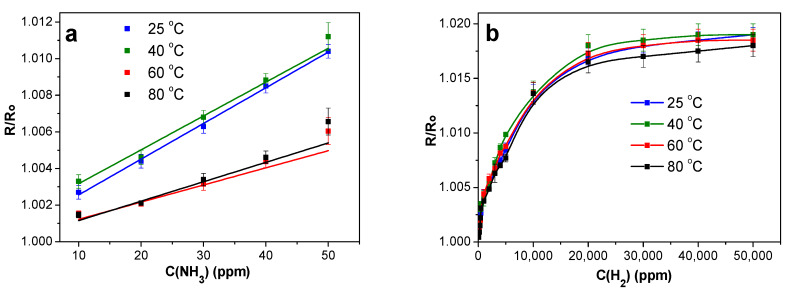
(**a**) Dependence of the sensor response of SWCNT/SiPc layers on concentration of NH_3_ and (**b**) H_2_ measured at different temperatures at RH 5%.

Because every sensor has its own preferred operating temperature, the sensor response of SWCNT/SiPc material toward ammonia and hydrogen was tested at 25, 40, 60 and 80 °C (Figure 12). The value of sensor response to both gases was maximal at 40 °C, however further temperature growth led to its decrease compared to that measured at room temperature. This behavior appears to be due to two competing processes, namely an increase of desorption [74] and growth of the concentration of charge carriers in p-type semiconductors with temperature [75]. Different rate of desorption of the chemisorbed oxygen from the surface of hybrid material at different temperatures may also contribute to these processes. Therefore, the operation temperature is a unique characteristic of each sensor. The maximal sensor response to ammonia is exhibited at 40 °C but the further increase of the temperature to 60–80 °C leads to the noticeable decrease of the response (Figure 12a). Similar tendency is also observed in the case of hydrogen detection (Figure 12b), however, the change is not as significant as in the case of ammonia.

## 3. Materials and Methods

### 3.1. Materials

Propargyl alcohol, dichlorosilicon (IV) phthalocyanine, copper(II) sulphate pentahydrate, sodium-L-ascorbate, NaN_3_, iodine monochloride and all solvents were purchased from Sigma Aldrich (Saint Louis, MO, USA). All reactions were carried out under a dry argon atmosphere unless otherwise noted. Column chromatography for the purification of the crude compounds was performed on silica gel 60. The purity of the products was tested at each step using thin layer chromatography (silica gel F-254 coated TLC plates).

### 3.2. Synthesis

#### 3.2.1. Azido Functionalized SWCNTs (SWCNT–N3)

Azido functionalized SWCNTs (SWCNT–N3) were prepared according to the literature [58]. For this purpose, 250 mg (3.84 mmol) NaN_3_ and 165 mg (1.01 mmol) iodine monochloride were dissolved in 25 mL of acetonitrile under a nitrogen atmosphere. This mixture was magnetically stirred for 15 min at 0 °C, followed by adding 20 mg SWCNTs to the solution; the reaction was stirred at room temperature for 24 h and filtered off. The solid products were washed with DMF and ethanol to remove unreacted compounds, then dried under vacuum.

#### 3.2.2. Axially-bis(propynoxy)phthalocyaninato Silicon (IV) (SiPc)

Axially-dichlorophthalocyaninato silicon (IV) (200 mg, 0.32 mmol) and propargyl alcohol (0.376 mL, 6.4 mmol) were refluxed in dry pyridine (15 mL) under an argon atmosphere for 48 h. Then the reaction mixture was cooled to room temperature and poured into water. The resulting solid was filtered and washed several times with hot water. The crude product was purified by column chromatography using a mixture of chloroform-EtOH (99:1) solvents as eluent. A green solid product was obtained and dried over phosphorus pentoxide. Yield: 30 mg (31%), Chemical formula: C_38_H_22_N_8_O_2_Si; FT-IR (attenuated total reflection (ATR), cm^−1^): 3261 (C≡C-H), 3010 (Ar-CH), 2957–2852 (Aliphatic CH), 2110 (C≡C), 1124 (Si-O), 1081 (C-O); UV/Vis (chloroform) λ_max_/nm (log ε): 672 (4.76), 606 (3.95), 354 (4.28); ^1^H-NMR (500 MHz, CDCl_3_) δ ppm: 9.68–9.66 (m, 8H, Pc-H_α_), 8.37–8.36 (m, 8H, Pc-H_β_), 0.63 (s, 2H, acetylene-H), -0.99 (s, 4H, -CH_2_), MALDI-TOFF (DHB): 650 [M]^+^, 595 [M-OCH_2_CCH]^+^; Anal. Calc. for C_38_H_22_N_8_O_2_Si; C 70.14; H 3.41; N 17.22%; Found: C 70.09; H 3.38; N 17.12%.

#### 3.2.3. Preparation of the Hybrid SWCNTs Nanomaterial by Functionalization with Silicon (IV) Phthalocyanine (SWCNT/SiPc)

The synthetic pathways of axially terminal propynoxy substituted silicon (IV) phthalocyanine (SiPc) and its hybrid nanomaterial with SWCNTs are presented in Figure 1.

Azido-substituted single walled carbon nanotubes (SWCNT-N3, 5 mg) were placed in a 10 mL reaction flask, and dimethylformamide (DMF, 3 mL) was added. The suspension was then sonicated for 30 min. Separately, SiPc (20 mg, 0.031 mmol) bearing axially terminal propynoxy groups was dissolved in DMF (2 mL). The SiPc solution was added to the SWCNT-N3 suspension dropwise. Copper(I) catalyst was prepared by mixing copper(II) sulfate pentahydrate (0.225 mg, 0.0009 mmol) and sodium L-ascorbate (0.90 mg, 0.0045 mmol) in distilled water (1 mL). This catalyst was added to the reaction mixture and the mixture was kept at 90 °C overnight. Finally, the reaction mixture was washed with water, ethanol, acetone and dried in a vacuum oven at 50 °C. FT-IR (ATR, cm^−1^): 3030–3066 (Ar-CH), 2990–2835 (aliphatic CH), 1518 (C=C), 1135 (Ar C=C), 1122 (Si-O).

### 3.3. Characterization Methods

The structure of axially-bis(propynoxy)phthalocyaninato silicon (IV) was characterized by the methods of elemental analysis (Flash 1112 Instrument, Thermo Finnigan, Waltham, MA, USA), MALDI mass-spectrometry (Matrix Assisted Laser Desorption Ionization (MALDI) Microflex LT, Bruker, Bruker Daltonics MS, Bremen, Germany), ^1^H-NMR (500 MHz spectrometer, Varian, California, USA, tetramethylsilane was an internal standard), FTIR (Spectrum 100 spectrophotometer, Perkin Elmer, Waltham, MA, USA) and UV–vis spectrophotometry (PG Instruments, Lutterworth, UK or Shimadzu 2101, Kyoto, Japan) in the range of 190–1100 nm. The composition of the novel hybrid material was confirmed by FT-IR and Raman (a LabRAM Horiba single spectrometer equipped with a CCD Symphony detector (Jobin Yvon, HORIBA, Montpellier, France), 488 nm, 20 mW line of Ar-laser) spectroscopies. All vibrational spectra are given without background subtraction. The morphology of the obtained hybrid was investigated via scanning electron microscopy (SEM) using a FEI-Nova system (Hillsboro, OR, USA) with magnification of 160,000× and high voltage of 10 kV and transmission electron microscopy (TEM) (G2 F20S-TWIN instrument, a versatile multipurpose 200 kV STEM/TEM/nano-analysis system, Tecnai, Hillsboro, OR, USA). Thermogravimetric analysis (TGA) was carried out using a STAReThermal Analysis System (Mettler Toledo, Zurich, Switzerland) at the heating rate of 10 °C∙min^−1^ under a nitrogen flow (50 mL·min^−1^).

### 3.4. Sensor Properties

The chemiresistive sensor response of the novel hybrid material toward different gases (NH_3_, CO_2_, H_2_S, H_2_ and their mixtures) was studied as described in the literature [37,76,77,78]. Hybrid suspensions (20 µL, 2 mg·mL^−1^) in dichloromethane subjected to ultrasonic treatment were spun onto a glass substrate with interdigitated Pt electrodes having the 10 μm dimension gap (G-IDEPT10; cell constant is 0.0118 cm^−1^ DropSens, Oviedo, Spain). Then a substrate connected with an electrometer (mod. 236, Keithley, Beaverton, OR, USA) was placed into the flow cell with the internal volume of 5 cm^3^. The resistance of layer was constantly measured at the dc voltage of 10 V. Analyzed gases diluted by dry air to the required concentration were passed through the cell with the constant flow rate of 300 mL∙min^−1^ during 30 s. Then the gas flow was blocked, and the layer was held until its resistance reached a steady state value. After that, the cell was purged with air. The next portion of gas was supplied after the resistance of the film reached its initial value. The relative sensor response was calculated as R/R_o_, where R is the current layer resistance at the detected concentration of the analyte and R_o_ is the layer resistance before the analyte injection. For the investigation of humidity effect, the measurements were carried out in the same way but the carrier gas (air) was bubbled through distilled water. The relative humidity (RH) inside the cell was monitored by a humidity meter (MPE-202.013).

## 4. Conclusions

The novel hybrid nanomaterial (SWCNT/SiPc) made of of SWCNTs cross-linked by axially substituted silicon (IV) phthalocyanine (SiPc) was obtained by the azide-alkyne cycloaddition (Click) reaction of azido-substituted SWCNTs with axially-bis(propynoxy)phthalocyaninato silicon (IV) (SiPc). The synthesized axially bis(propynoxy) substituted silicon (IV) phthalocyanine and its hybrid were characterized by spectral methods (FT-IR, ^1^H-NMR, UV-Vis, mass), thermogravimetry and electron microscopy (SEM and TEM). According TGA data, the amount of linking SiPc molecules covalently bonded to SWCNT was estimated to be one SiPc molecule per 57 carbon atoms. The layers of the prepared hybrid were tested as active layers of chemiresistive sensors toward ammonia (1–50 ppm) and hydrogen (100–60,000 ppm) at different temperatures, relative humidity and in the presence of interfering gases like carbon dioxide, hydrogen sulfide and volatile organic vapors. The hybrid nanomaterial exhibited the completely reversible sensor response to both gases at room temperature; the recovery time was 100–200 s for NH_3_ and 50–120 s in the case of H_2_ depending on the gas concentrations. At RH 20% the sensor response was almost the same as that measured at RH 5%, whereas the further increase of RH to 50 and 70% led to its 2–3 fold decrease. It was demonstrated that the SWCNT/SiPc layers can be successfully used for the detection of both NH_3_ and H_2_ in the presence of CO_2_. On the contrary, H_2_S was found to be an interfering gas for the NH_3_ detection. 

## Figures and Tables

**Table 1:** Sensor characteristics of active layers based on carbon nanomaterials functionalized with metal oxides, phthalocyanines, and polymers.

NH_3_ Sensing Layer	LOD, ppm	Linear Range, ppm	Temp. Range, °C	Ref.
SWCNT/pyrene-3D	0.5	0.5–5	RT	[45]
GO/CoPc	0.8	0.8–50	RT	[64]
rGO/MPc	0.8	0.8–50	RT	[70]
G/PEDOT-PSS	10	5–20	RT	[71]
MWCNT/CuPc	0.75	0.6–5; 10–30	RT	[72]
SWCNT/SiPc	0.5	0.5–50	25–80	This work
**H_2_ Sensing Layer**	**LOD, ppm**	**Linear Range, ppm**	**Temp. Range,** **°C**	**Ref.**
SWCNT/SnO_2_	100	100–10,000	RT	[67]
SWCNT/Pd	50	50–500	RT	[68]
SWCNT/In_2_O_3_, ZnO or SnO_2_	500	500–2000	25–200	[73]
SWCNT/SiPc	70	70–1000	25–80	This work
